# Dysbindin facilitates invasion and metastasis by promoting phosphorylation of ERK in epithelial ovarian cancer

**DOI:** 10.7150/jca.39269

**Published:** 2020-02-21

**Authors:** Xiaohui Lv, Xin Guo, Yi Ru, Fuxing Zhou, Xiaoshan Yang, Junli Ge, Jia Li, Shujuan Liu, Kuo Jiang, Biliang Chen

**Affiliations:** 1 Department of Gynecology and Obstetrics, Xijing Hospital, Fourth Military Medical University, Xi'an, Shaanxi 710032, China.; 2 Department of General Surgery, Chinese People's Liberation Army General Hospital, Beijing 100853, China.; 3 Department of Endoscopic Surgery, Chinese People's Liberation Army 986 th Hospital, Fourth Military Medical University, Xi'an, Shaanxi 710054, China.; 4 Department of biochemistry and molecular biology, Fourth Military Medical University, Xi'an, Shaanxi 710032, China.; 5 State Key Laboratory of Military Stomatology & National Clinical Research Center for Oral Diseases & Shaanxi International Joint Research Center for Oral Diseases, Center for Tissue Engineering, School of Stomatology, Fourth Military Medical University, Xi'an, Shaanxi 710032, China.; 6 Department of Spine Surgery, Honghui Hospital, Xi'an Jiaotong University College of Medicine, Xi'an, Shaanxi 710054, China.

**Keywords:** Dysbindin, EMT, ERK, Invasion, Metastasis

## Abstract

Dysbindin has been reported to be correlated with several malignancies. However, the clinical significance and biological role of dysbindin in epithelial ovarian cancer remains largely unknown. Here we demonstrated that the mRNA and protein levels of dysbindin were significantly upregulated in EOC tissues compared with normal ovarian tissues. High levels of dysbindin were significantly correlated with worse clinicopathological characteristics and poor prognosis in EOC patients. Overexpression and silencing of dysbindin promoted and inhibited, respectively, invasion and metastasis of EOC cells *in vitro* and* in vivo*. Mechanistically, dysbindin activates phosphorylation of ERK to promote the epithelial-mesenchymal transition and to mediate the invasive and metastatic ability of EOC cells. Our findings suggest that dysbindin facilitates invasion and metastasis by promoting the EMT of EOC cells and may serve as a potential therapeutic target in EOC.

## Introduction

Epithelial ovarian cancer (EOC) ranks for the fourth leading cause of cancer-related deaths worldwide 1,2. Patients with EOC exhibited poor prognosis due to the lack of effective diagnostic and prognostic biomarkers 3. Although mounting efforts have been made, the outcomes of EOC patients have not improved substantially. Thus, searching for novel biomarkers which illuminated the progression and prognosis of EOC is urgently required.

Dysbindin (DTNBP1), a novel oncoprotein, has been reported to be a regulator of Akt signaling pathway 4. Dysbindin has been demonstrated to be ubiquitously distributed in central nervous system and embryonic tissues 5. Since many tissues regained the ability to proliferate during the embryo when they were turned to cancer tissues, like liver cancer secreting AFP and CT genes in testicle cancer 6,7. It is speculated that dysbindin which mainly functions in embryonic period may likely be involved in functions of cancers 8. Importantly, dysbindin has been identified to be dysregulated in various cancers including melanoma 9 and oral squamous cell carcinoma 10. In these cancers, dysbindin serves as an oncoprotein participating in tumor invasiveness and progression and significantly correlated with patients' prognosis. In a recent study, dysbindin showed significant correlation with pancreatic cancer and promoted tumor proliferation *in vitro* and *in vivo* 4, 11. Thus, improved knowledge of functions in dysbindin may help understand biology and provide optimal strategies in human cancers.

The role and clinical significance of dysbindin remain largely unclear in EOC. In this study, we firstly assessed the expression levels of dysbindin in EOC tissues and normal ovarian tissues and evaluated the relationship with clinical characteristics. Then, we investigated the prognostic value of dysbindin. Finally, we revealed that dysbindin enhanced cell invasion and EMT-like features by promoting phosphorylation of ERK.

## Materials and methods

### Tissue samples

The 104 EOC and 36 normal ovarian samples used for RT-PCR and Western blot were obtained in Xijing Hospital. The EOC tissue specimens were harvested from EOC patients who underwent primary surgery, and the normal ovarian specimens were obtained from patients who underwent either hysterectomy or adnexectomy due to cervical intraepithelial neoplasia. The additional cohort including 226 EOC samples were surgically obtained between June 2013 and June 2016. Every single EOC sample was prepared for five 4-μm sections. The histological staging of EOC patients was based on the International Federation of Gynecology and Obstetrics (FIGO) criteria. The ethics committee of Xijing Hospital has already approved this study.

### Immunohistochemistry (IHC)

The immunohistochemistry staining procedures were adopted, as was previously introduced 4. Three independent pathologists firstly evaluated the H&E staining sections to confirm whether the specimens were EOC tissues. And then the immunohistochemical evaluations were performed. The staining score was determined with the following formula: intensity score × proportion score. Briefly, the IHC intensity was defined as follows: negative, 0; weak, 1; moderate, 2; strong, 3 4. The IHC proportion scores were defined as follows: 0%, 0; 0.01-25%, 1; 25.01-50%, 2; 50.01-75%, 3; >75%, 4 12. The staining score of each section was graded as follows: -, 0, negative; +, 1-4, weak positive; ++, 5-8, moderate positive; +++, 9-12, strong positive 12. Low expression levels were considered as staining score of “-”and “+”, and high expression levels represented “++” and “+++”, as previously reported 4.

### RNA extraction and Real-Time Polymerase Chain Reaction

Trizol was used to extract the total RNAs. Reverse transcription was carried out with RT-PCR kit (Takara) based on product manuals. Using β-actin as house-keeping gene, the RT-PCR cycling was performed as follows: 95 °C for 1min, 45 cycles of 95 °C for 5 sec, and 60 °C for 30 sec. All the reactions were carried out in triplicate. The primers synthesized by Sangon Biotech were the following sequences which were reported previously 11. The commonly used 2^-ΔΔCt^ method was performed to assess the target genes' expression 13.

### Western blot

Proteins from tissues or cells were extracted with strong RIPA buffer (GenePharma) which included protease and phosphatase inhibitors. Afterwards, the lysates were re-cracked with sodium dodecyl sulfate (SDS) prior to polyacrylamide gel electrophoresis. When transferred to a polyvinylidene fluoride (PVDF) membrane, the gel was pre-incubated with corresponding antibodies in advance. A secondary antibody comprising a peroxidase-conjugated anti-mouse or anti-rabbit antibody was used as indicated. Finally, chemiluminescence assays were performed to visualize the bands. Proteins were normalized to GAPDH and quantified with ImageJ software.

### Cell culture

Human EOC cell lines including SKOV3, A2780, CAOV3 and 8910, human embryonic kidney cells 293T, and human pancreatic ductal adenocarcinoma cells PANC-1, Aspc-1 and CFPAC-1 were obtained from Shanghai cellular biology center. All cells were cultured at 37℃ with 5% CO_2_ with DMEM or RPMI-1640 containing 10% fetal bovine serum, 100 U/mL penicillin and 100 μg/mL streptomycin. Cells involved in all the experiments were carried out within 5 passages.

### Plasmids construction and viral transfection

To interfere with dysbindin expression, two different human dysbindin-targeting siRNA sequences, namely dysbindin-RNAi-1 and dysbindin-RNAi-2, were cloned to generate dysbindin-shRNA(s) with the sequences as previously reported 11 (synthesized by GenePharma). A scrambled shRNA was synthesized and inserted into dysbindin to generate the negative control. Target cells were transfected with oligonucleotides using the Lipofectamine 2000 reagent (Invitrogen, Carlsbad, CA) according to the manufacturer's instructions. The infected cells were selected for stable cell lines with puromycin (1.0 μg/mL) for 10 days.

To enhance the expression of dysbindin, a plasmid inserted with a PCR-amplified human dysbindin cDNA was user to establish the dysbindin overexpression cell line. The empty vector was used to transfected the control cell line. Target cells were also transfected with oligonucleotides using the Lipofectamine 2000 reagent (Invitrogen, Carlsbad, CA) according to the manufacturer's instructions. The final stable cell lines were selected with puromycin (1.0 μg/mL) for 10 days. The resulting cells used for following experiments were not with the addition of puromycin.

### *In vitro* invasion and migration assays

The upper transwell chamber was precoated with Matrigel, and EOC cells of 1 × 10^5^ were seeded in 200 μl medium without fetal bovine serum. 400 μl medium with fetal bovine serum were added into lower chamber. Then the transwell chambers with cells were cultured for 36h. After fixing with 95% ethanol and stained with crystal violet for 15min, the upper chambers were washed with PBS for three times. Finally, the lower-side of the chamber was counted for cell numbers with microscope.

Wound healing assays were performed to check cell migration speed. Cells of 1 × 10^5^ were incubated in a 6-well plate overnight. The plate was scratched with a straw tip and pictured at 0 h and 24 h later. The distance migrated by the cell monolayer to close the scratch area during the time period was observed and measured.

### Xenograft tumor models

The Balb/c athymic nude female mice (6 weeks of age) were kept under specific pathogen-free conditions (12 h light and dark cycles), with food and water supplied ad libitum. The animal protocols were approved by the Institutional Animal Care and Use Committee of Xijing Hospital. The animals for *in vivo* metastasis assays were injected with 1 × 10^7^ cells of transfected and control cells intraperitoneally 14. All mice were observed regularly and sacrificed before natural death occurred. Tumor nodules were removed, counted, and the mice were weighed.

### Statistical analysis

SPSS 19.0 and Graphpad PRISM software was used in this study. Mean ± standard deviation (SD) was adopted to show all the data unless specially noted. Two independent groups were analyzed with Student's t test. Three or more groups were analyzed with ANOVA test. Kaplan-Meier curves were plotted to estimate the patients' overall survival (OS). A value of <0.05 was considered statistical difference.

## Results

### Dysbindin exhibits increased expression levels in EOC tissues

**Figure 1:**
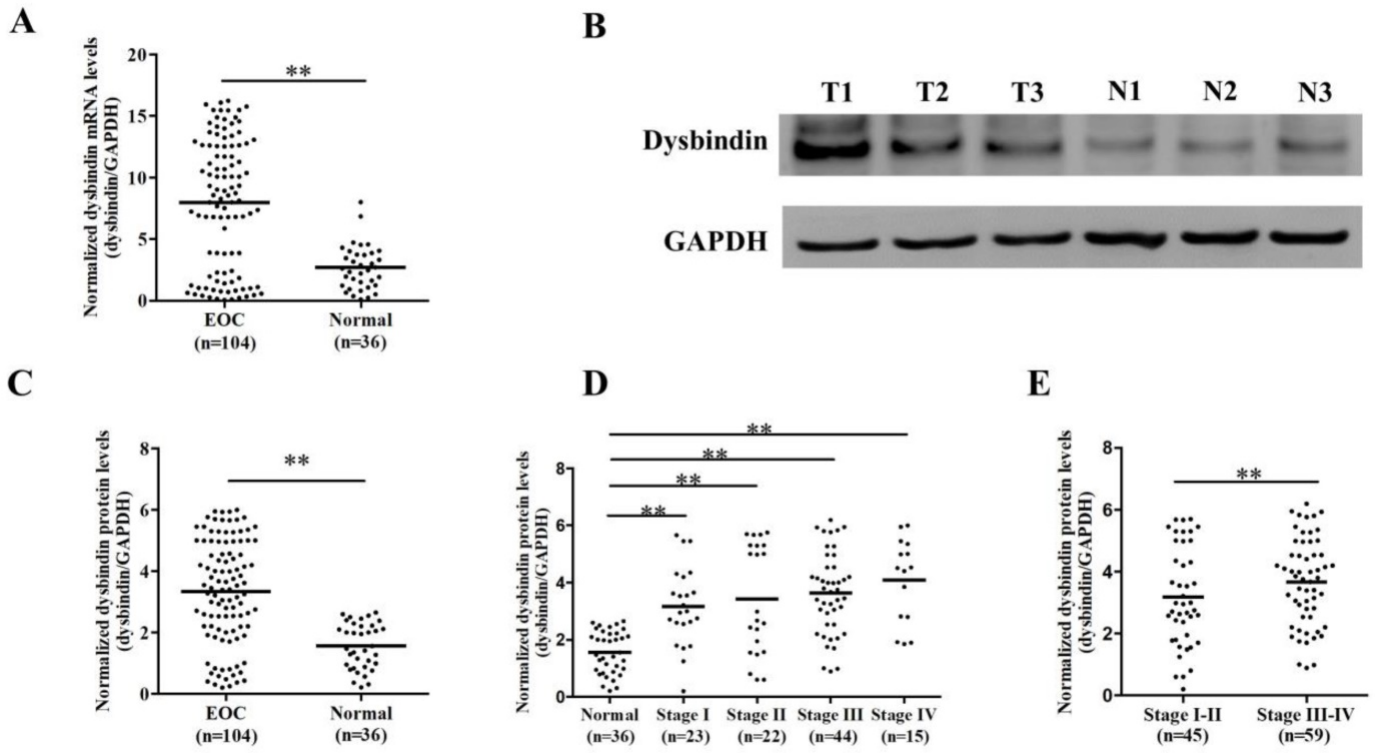
** (A)** Scatter plot of dysbindin mRNA expression confirmed by RT-PCR in EOC and normal ovarian tissues. EOC: epithelial ovarian cancer; Normal: normal ovarian tissue. **: P < 0.001. **(B)** Representative bands of dysbindin protein expression confirmed by Western blot in EOC and normal ovarian tissues. T: ovarian cancer tissue; N: normal ovarian tissue. **(C)** Scatter plot of dysbindin protein expression normalized to GAPDH in EOC and normal ovarian tissues. **: P < 0.001. **(D-E)** Scatter plot of dysbindin protein expression normalized to GAPDH in EOC of different stages. **: P < 0.001.

Firstly, we enrolled a validation group including 104 EOC and 36 normal ovarian tissues and assessed the expression levels of dysbindin with RT-PCR and Western blot, respectively. The clinical characteristics of patients in validation group were presented in Table 1. Dysbindin mRNA expression was significantly upregulated in samples from EOC tissues compared with normal ovarian ones (P<0.001, Fig. 1A), and dysbindin protein expression levels were also dramatically increased in tumor tissues than normal ovarian ones (P<0.001, Fig. 1B and C). In addition, the expression levels of dysbindin at all stages (I to IV) were all higher than those of normal tissues (all P<0.001, Fig. 1D). Particularly, the expression levels of dysbindin were upregulated in stage III-IV EOC tissues than those of stage I-II cancers (P<0.001, Fig. 1E). Taken together, dysbindin exhibited high levels of mRNA and protein in EOC samples, highlighting its potential oncogenic role during the advanced stages of EOC development.

### Upregulation of dysbindin in tumor is associated with poor prognosis in EOC

**Figure 2:**
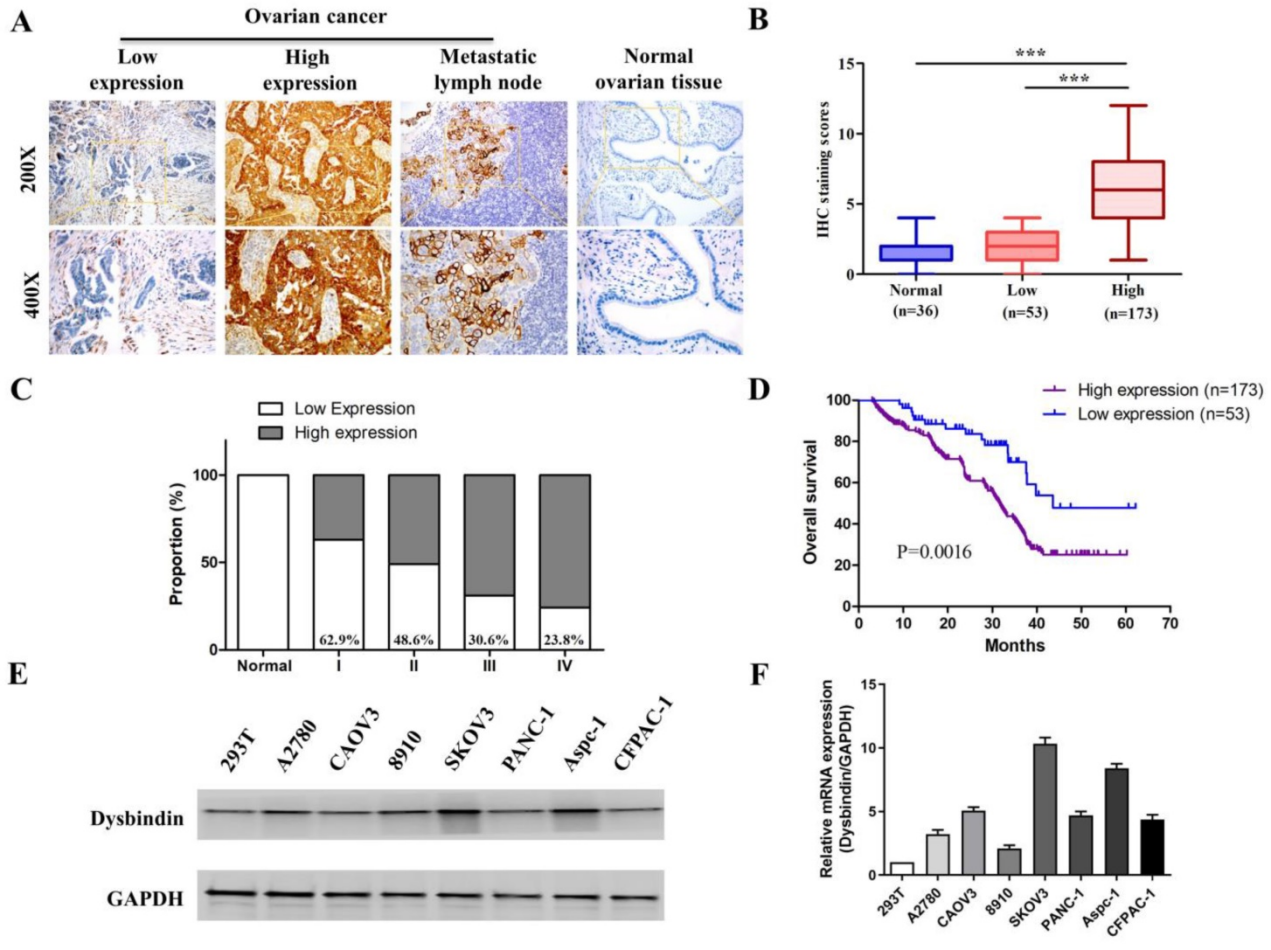
** (A)** Representative IHC staining of dysbindin in EOC and normal ovarian tissues. **(B)** IHC staining scores of normal ovarian tissues, dysbindin low expression group and high expression group. ***: P < 0.0001. **(C)** The proportions of dysbindin high expression tissues in normal ovarian tissues and different stage EOC tissues.** (D)** Overall survival of patients with high and low dysbindin expression. **(E)** Representative bands of dysbindin protein expression confirmed by Western blot in EOC cells.** (F)** Dysbindin mRNA expression confirmed by qRT-PCR in EOC, pancreatic ductal adenocarcinoma cells. Low expression levels were considered as staining score of “-”and “+”, and high expression levels represented “++” and “+++”.

Next, we performed IHC in an additional 226 primary EOC samples which were assigned as “verification group” to further confirm the significance of dysbindin expression. The clinical characteristics of patients in validation group were presented in Table 1. Dysbindin staining was low or absent in normal ovarian tissues but was high in ovarian cancer and metastatic lymph node (Fig. 2A). EOC tissues were classified into two groups based on dysbindin expression levels: 173 of the 226 (76.5%) samples presented high expression levels of dysbindin and were classified as “dysbindin high expression group”; 53 of the 226 (23.5%) samples presented low or undetectable expression levels of dysbindin and were classified as “dysbindin low expression group” (Fig. 2B). Furthermore, the proportion of dysbindin high expression in ovarian cancer tissues was increased with clinical stage (Fig. 2C).

Then, the prognostic value of dysbindin ovarian cancer was evaluated. Kaplan-Meier survival analysis indicated that patients with high dysbindin expression exhibited shorter OS (HR=1.768, 95%CI (1.253-2.308), P<0.001) than those with low dysbindin expression (Fig. 2D). In addition, patients with high dysbindin expression showed more deleterious clinical features including FIGO stage (P=0.026), lymph node metastasis (P=0.017) and ascites (P=0.013, Table 2). Univariate and multivariate analysis both indicated that dysbindin expression was an independent prognostic factor for OS (P=0.001, Table 3). These results suggest that dysbindin may exacerbate the progression of EOC and serve as a promising prognostic marker.

### Dysbindin promotes tumor invasion and metastasis *in vitro and in vivo*

**Figure 3:**
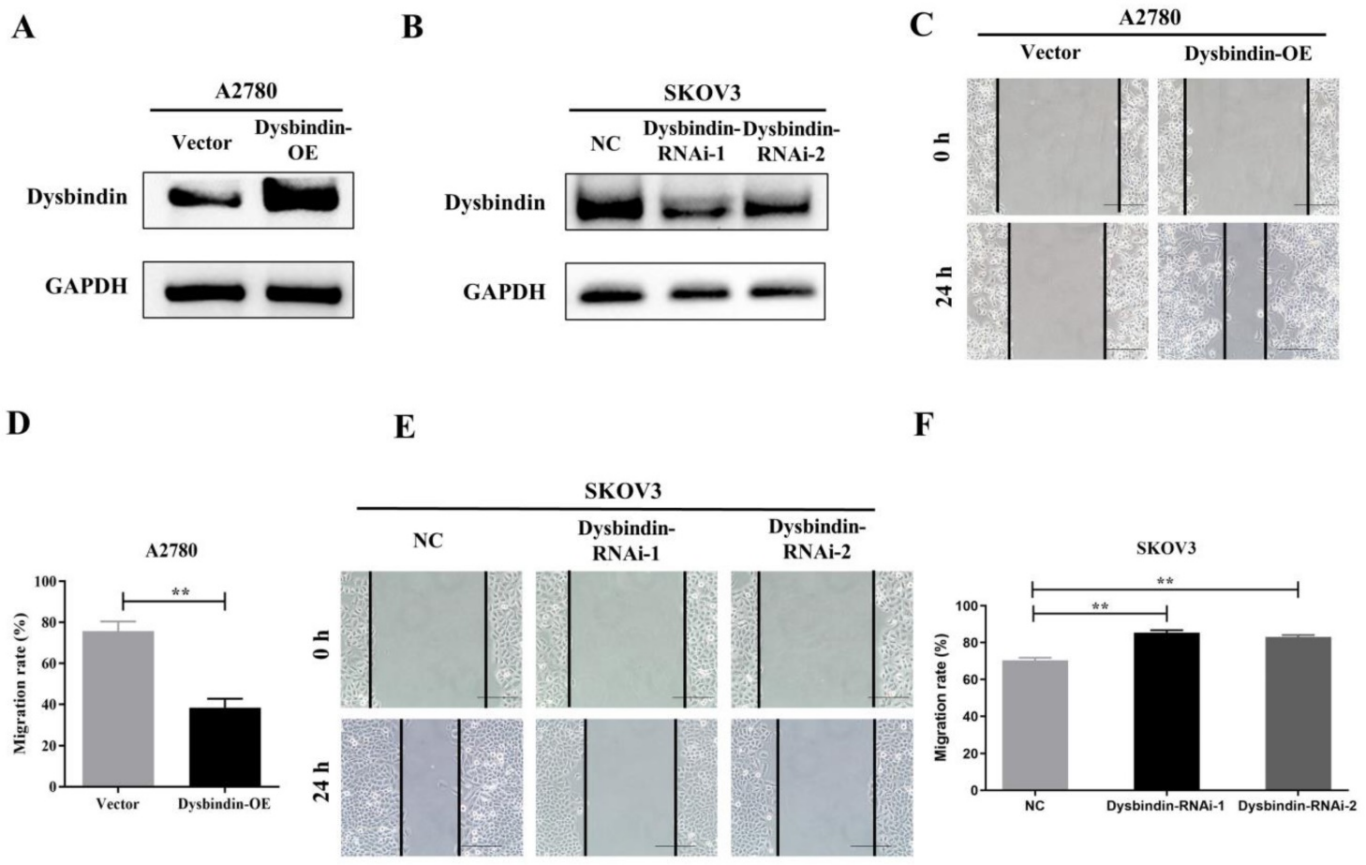
** (A-B)** RT-PCR and Western blot identified the inhibition of dysbindin in SKOV3 cell line and the overexpression of dysbindin in A2780 cell line. NC: negative control; OE: overexpression. **(C)** Detection of migration speed of cancer cells at indicated time in A2780-Vector and A2780-dysbindin-OE cell lines. N=3 cells. **(D)** Quantitative analysis of above-mentioned migrated cells. Data with error bars represent mean ± s.e.m. **: P < 0.001. **(E)** Detection of migration speed of cancer cells at indicated time in SKOV3-NC, SKOV3-dysbindin-RNAi-1 and SKOV3-dysbindin-RNAi-2 cell lines. N=3 cells. **(F)** Quantitative analysis of above-mentioned migrated cells. Data with error bars represent mean ± s.e.m. **: P < 0.001.

As dysbindin is previously reported to be involved in material transportation in nerve cells of mouse embryos which indicated a potential role in cell movement 15, 16 and our clinical data indicated dysbindin overexpression correlated with metastatic potential, we postulated that dysbindin exhibited potential effects on tumor invasion and metastasis. To prove this hypothesis, we firstly evaluated the mRNA and protein expression of dysbindin in 4 different EOC cells lines including A2780, CAOV3, 8910, and SKOV3. Expressions of dysbindin were enriched in more invasive cell lines like SKOV3 17 and lessened in less invasive cell lines like A2780 18 which were reported with high and low metastatic potential (Fig. 2E and 2F). Thus, we chose A2780 and SKOV3 of which the expression levels of dysbindin were low and high for further experiments. To investigate the functional role of altered dysbindin expression in EOC and whether dysbindin modulates the invasive behaviors of the EOC cells, we established stable dysbindin-OE (overexpression) A2780 cell lines and dysbindin-knockdown SKOV3 cell lines. As shown in Fig. 3A and 3B, we successfully established dysbindin-RNAi-1, dysbindin-RNAi-2, dysbindin-RNAi-NC (negative control) in SKOV-3 cell line and dysbindin-OE and dysbindin-vector in A2780 cell line.

**Figure 4:**
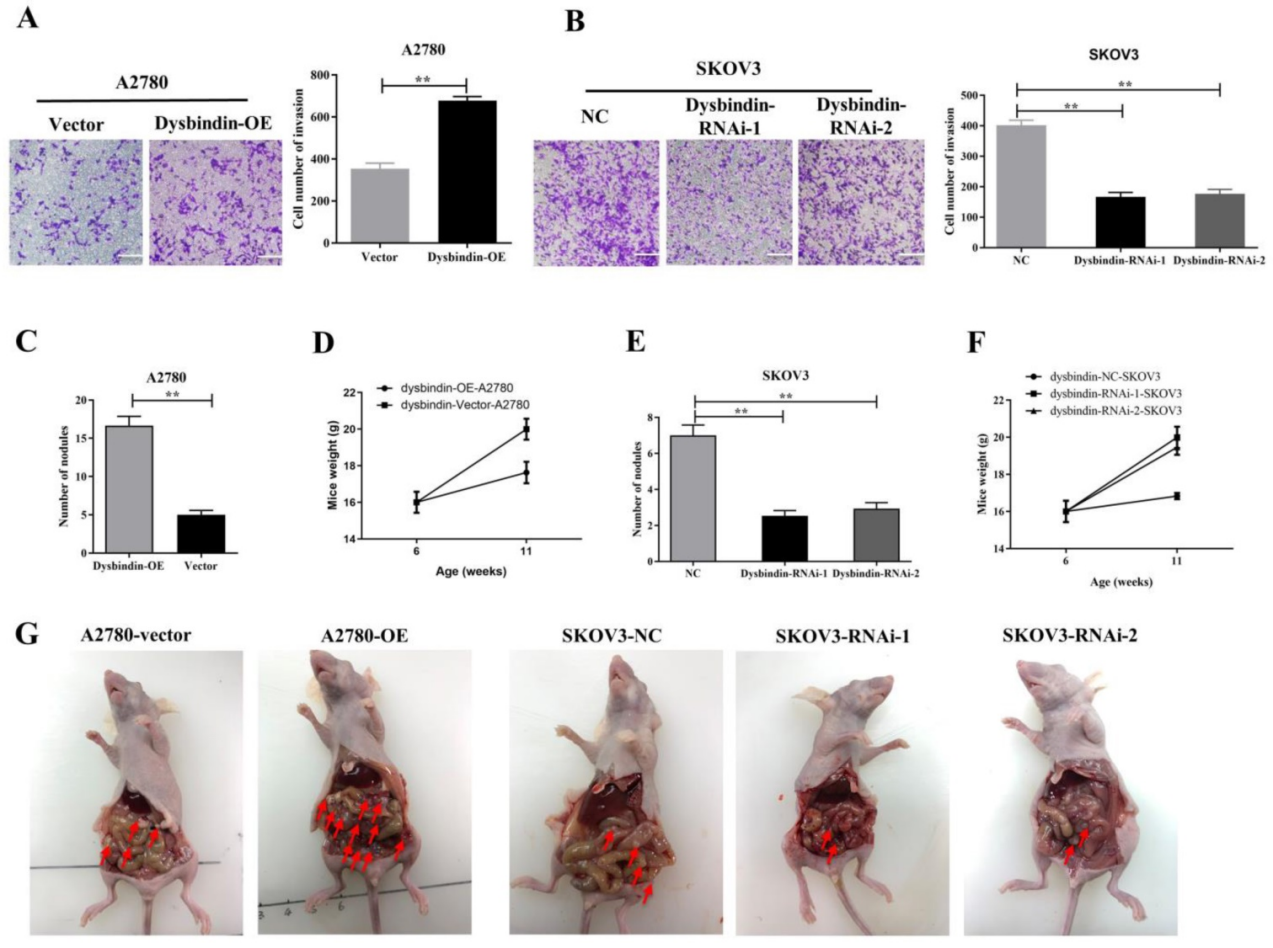
** (A)** Microscopy images and quantification of migrated cells by A2780-Vector and A2780-dysbindin-OE cell lines after incubation in permeable supports for 36 h, staining with crystal violet (magnification, 20×). Data with error bars represent mean ± s.e.m. **: P < 0.001. **(B)** Microscopy images and quantification of migrated cells by SKOV3-NC, SKOV3-dysbindin-RNAi-1 and SKOV3-dysbindin-RNAi-2 cell lines after incubation in permeable supports for 36 h, staining with crystal violet (magnification, 20×). Data with error bars represent mean ± s.e.m. **: P < 0.001. **(C)** Number of nodules extracted from mice' peritoneal cavity generated by A2780-Vector and A2780-dysbindin-OE cell lines. **: P < 0.001. n=6 mice/group. **(D)** Mice weights injected with A2780-Vector and A2780-dysbindin-OE cell lines were measured before injected and euthanized, respectively. n=6 mice/group. **(E)** Number of nodules extracted from mice' peritoneal cavity generated by SKOV3-NC, SKOV3-dysbindin-RNAi-1 and SKOV3-dysbindin-RNAi-2 cell lines. **: P < 0.001. n=6 mice/group. **(F)** Mice weights injected with SKOV3-NC, SKOV3-dysbindin-RNAi-1 and SKOV3- dysbindin-RNAi-2 cell lines were measured before injected and euthanized, respectively. n=6 mice/group. **(G)** The general pictures of xenograft tumor models. Arrows indicates as peritoneal metastatic nodules.

Then, we performed transwell and would healing assays. Wound healing assays showed dysbindin overexpression significantly promoted cell motion in dysbindin-OE A2780 cells and impaired cell motion in dysbindin-knockdown SKOV3 cells (all P<0.001, Fig. 3C-3F). Similarly, transwell assays showed that cell invasion was enhanced in dysbindin-OE A2780 cells compared with dysbindin-vector cells (both P<0.001, Fig. 4A). Inversely, dysbindin knockdown was correlated with a dramatical inhibition in cell invasion (all P<0.01, Fig. 4B). Furthermore, we performed animal assays to evaluate dysbindin on cancer metastasis *in vivo*. Immunodeficient nude mice were intraperitoneally injected with dysbindin-OE A2780 cells or dysbindin-knockdown SKOV3 cells. As shown in Fig. 4C and 4G, the number of metastatic nodules in peritoneal cavity which were injected with dysbindin-OE A2780 cells was more than those with dysbindin-vector A2780 cells. In addition, mice treated with dysbindin-OE A2780 cells lose more weight than those treated with dysbindin-vector A2780 cells (Fig. 4D). Inversely, mice treated with dysbindin-RNAi-1 or dysbindin-RNAi-2 cells presented a reduced number of metastatic tumor nodules and a heavier weight compared with those treated with dysbindin-RNAi-NC cells (Fig. 4E and 4F). These results confirmed the high expression levels of dysbindin in EOC cells and suggested that dysbindin promoted EOC cells invasion and metastasis *in vitro* and *in vivo*, supporting our observations in EOC clinical specimens.

### Dysbindin promoted epithelial-mesenchymal transition by activating ERK phosphorylation

**Figure 5:**
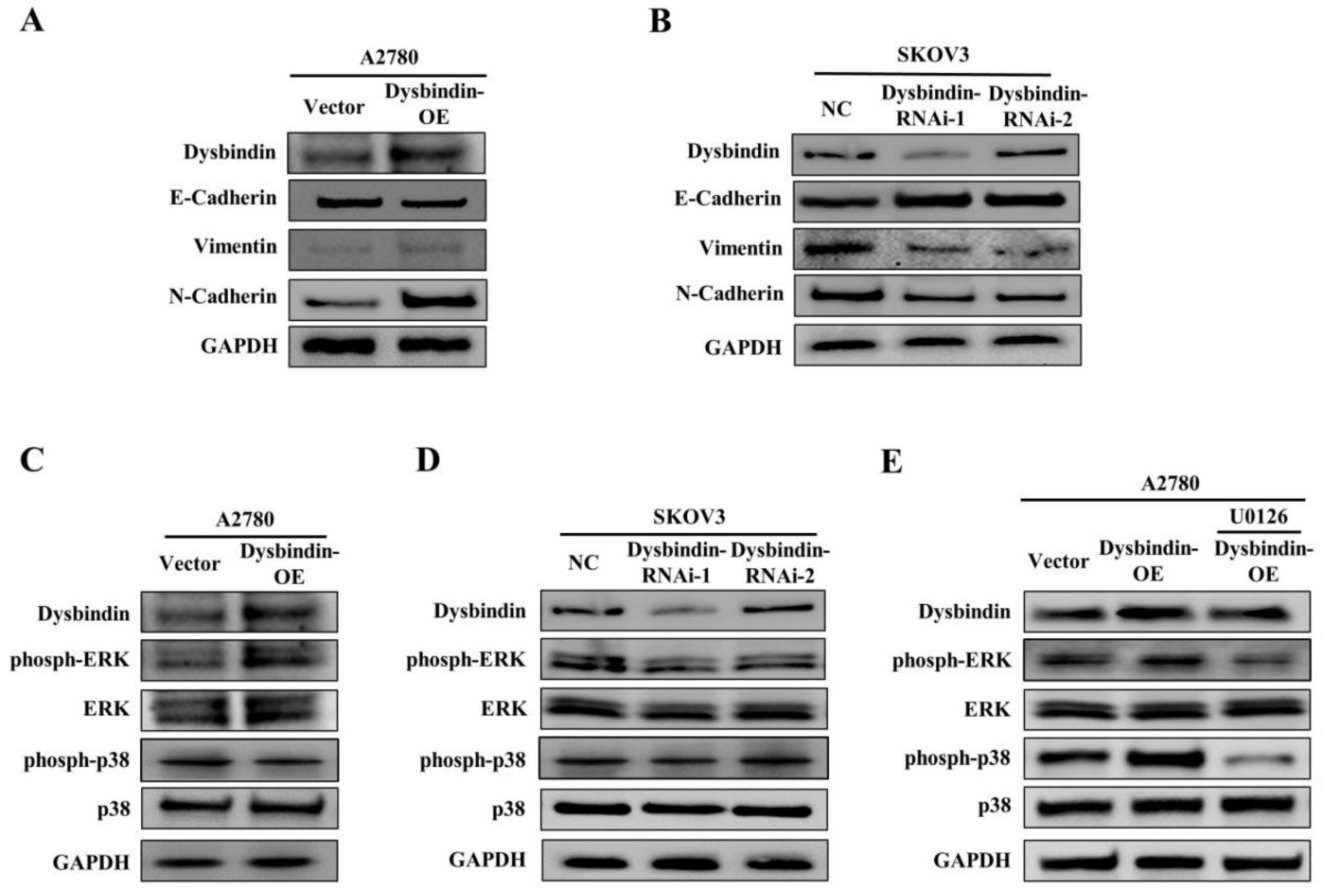
** (A)** Analysis of indicated proteins in A2780-Vector and A2780- dysbindin-OE cell lines. GAPDH is a loading control. **(B)** Analysis of indicated proteins in SKOV3-NC, SKOV3- dysbindin-RNAi-1 and SKOV3- dysbindin-RNAi-2 cell lines. GAPDH is a loading control. **(C)** Analysis of ERK-associated proteins in A2780-Vector and A2780- dysbindin-OE cell lines. GAPDH is a loading control. **(D)** Analysis of ERK-associated proteins in SKOV3-NC, SKOV3- dysbindin-RNAi-1 and SKOV3- dysbindin-RNAi-2 cell lines. GAPDH is a loading control. **(E)** Analysis of ERK-associated proteins in SKOV3-NC, SKOV3- dysbindin-RNAi-1 and SKOV3- dysbindin-RNAi-2 cell lines when treated with U0126. GAPDH is a loading control.

As tumor cell invasive and metastatic abilities represent crucial consequences of EMT, we further investigated the relationship between dysbindin and EMT in EOC cells. For the EOC transfected and control cells, we performed RT-PCR and Western blot to analyze the expression levels of EMT biomarkers, E-cadherin, N-cadherin and Vimentin. The results showed that the epithelial marker (E-cadherin) was up-regulated and the mesenchymal markers (N-cadherin and Vimentin) were down-regulated in dysbindin-RNAi-1 and dysbindin-RNAi-2 compared with dysbindin-NC SKOV3 cells (Fig. 5A). For dysbindin-overexpressed cells, the opposite molecular changes were observed (Fig. 5B). Furthermore, we found that the bioinformatics functional analysis of dysbindin revealed the potential role of promoting phosphorylation on ERK 8. Thus, we evaluated the affection of dysbindin knockdown on the activation of ERK and p38. Silencing of dysbindin significantly attenuated the phosphorylation of ERK while overexpression of dysbindin promoted the phosphorylation of ERK in A2780-OE and SKOV3-knockdown cells, respectively (Fig. 5C and D). However, when A2780-OE cells were treated with U0126 (the inhibitor of ERK phosphorylation), the phosphorylation levels of ERK did not change as dysbindin changed, implying that dysbindin was involved in ERK phosphorylation (Fig. 5E). Besides, the levels of phosph-p38 and p38 did not change with the expression of dysbindin, indicating that the Akt-involved p38 signaling pathway was not correlated with dysbindin in ovarian cancer. These results demonstrated that dysbindin overexpression induced a transition to mesenchymal phenotype in EOC cells. Taken together, these findings suggest that dysbindin promotes cancer cell invasion and metastasis by activating ERK phosphorylation and inducing EMT in EOC.

## Discussion

Ovarian cancer is one of the deadliest malignancies in gynecology, which seriously threatens the health of women. Since early symptoms of ovarian cancer are not obvious; and due to the lack of reliable early detection methods, nearly 80% of EOC patients have advanced tumors at the first diagnosis with extensive metastasis 2. According to the statistics, the five-year overall survival rate of EOC patients is less than 45% 19. Therefore, exploring the mechanism of recurrence and metastasis of ovarian cancer and finding the molecular regulatory network of metastasis are the key issues to improve the prognosis of ovarian cancer patients.

Dysbindin extensively participated in cellular morphology maintenance, material transportation and cell development in embryonic tissues and central nervous system since this protein was initially discovered in 2001 20. Higo et al. was the first one to identify the up-regulated levels of dysbindin in head and neck squamous carcinoma in 2005^10^. In pancreatic ductal carcinoma, dysbindin has been proved to be involved in tumor proliferation and correlated with poor prognosis 11. In neuroblastoma, dysbindin has been identified to modulate cell morphology by acting on actin and c-Jun signaling pathway was involved in this process 21. However, the role of dysbindin in epithelial ovarian cancer remains largely unknown.

In this study, we demonstrated that the high expression levels of dysbindin in EOC and lymph node metastatic tissues compared with normal ovarian tissues, and patients with high dysbindin expression had poor overall survival. These data support the hypothesis that dysbindin is a novel predictive biomarker for EOC and might correlate with tumor metastasis. Then, we manipulated the expression of dysbindin in two EOC cell lines and mainly investigate the effects of dysbindin on tumor invasion and metastasis. The results showed that dysbindin significantly promote EOC cells invasion and metastasis *in vitro* and *in vivo*, and induced EMT of EOC cells which were nominated as pre-metastatic state of cancer cells. Several studies have reported that EMT is emphasized for preparing for dissemination from primary tumors to the circulation system and even inducing tumor cell into a cancer stem cell like state. Thus, it is reasonable to speculate that dysbindin promoted EOC metastasis by inducing EMT.

However, although dysbindin may facilitate tumor cell invasion and metastasis by promoting EMT of EOC cells, the underlying mechanisms remain largely unknown. It is reported that dysbindin can enhance the phosphorylation of Akt-p38 and promote cell proliferation in pancreatic ductal adenocarcinoma 4. Another bioinformatics analysis indicated that dysbindin functions mainly in cell cycle and motion 8. In this study, we have found that dysbindin promoted the EMT of cancer cells by activating ERK phosphorylation and was not correlated with p38 in ovarian cancer. We guessed that dysbindin may function diversely in different cancers. Thus, the underlying molecular mechanisms of how dysbindin phosphorylate ERK were our focus in the future.

In summary, our study shed new lights that dysbindin promotes EOC cell invasion and metastasis by inducing epithelial-mesenchymal transition.

## Figures and Tables

**Table 1:** Clinical characteristics of validation and verification groups

Characteristics	Validation group		Verification group
	Ovarian cancer (%)	Normal ovarian tissue (%)	Ovarian cancer (%)
No. of patients	104	36	226
**Age (years)** <50 ≥50	44 (42.3) 60 (57.7)	15 (41.7) 21 (58.3)	101 (44.7) 125 (55.3)
**Histological grade** G1 G2 G3 Gx	8 (7.7) 3 (2.9) 73 (70.2) 20 (19.2)	- - - -	18 (8.0) 5 (2.2) 152 (67.3) 51 (22.6)
**Pathological differentiation** Serous Clear cell Mucinous Endometrioid	79 (76.0) 6 (5.8) 16 (15.4) 3 (2.9)	- - - -	168 (74.3) 13 (5.8) 29 (12.8) 16 (7.1)
**FIGO stage** I + II III + IV	45 (43.3) 59 (56.9)	- -	103 (45.6) 123 (54.4)
**Ascites** Negative Positive	18 (17.3) 86 (82.7)	- -	27 (11.9) 199 (88.1)
**Lymph node Invasion** Negative Positive	73 (70.2) 31 (29.8)	- -	143 (63.3) 83 (36.7)

**Table 2:** Correlations between dysbindin expression and clinical characteristics in patients with EOC

Characteristics	Dysbindin expression		χ^2^	*P* value
	High (n=173)	Low (n=53)		
**Age (years)**			0.423	0.513
<50	76	25		
≥50	97	28		
**Histological grade**			0.397	0.487
G1	15	3		
G2	4	1		
G3	123	29		
Gx	31	20		
**Pathological differentiation**			2.223	0.772
Serous	134	34		
Clear cell	9	4		
Mucinous	24	5		
Endometrioid	6	10		
**FIGO stage**			8.998	**0.026**
I + II	87	16		
III + IV	86	37		
**Ascites**			9.545	**0.017**
Negative	22	5		
Positive	151	48		
**Lymph node invasion**			11.232	**0.013**
Negative	116	27		
Positive	57	26		

**Table 3:** Univariate and multivariate analyses of prognostic parameters for OS in patients with EOC

Risk factors	Univariate OS analysis			Multivariate OS analysis		
	HR	95% CI	*P* value	HR	95% CI	*P* value
Dysbindin expression (high vs. low)	1.742	1.393-2.371	**<0.001**	1.682	1.293-2.291	**0.001**
Age (<50 vs. ≥50)	0.991	0.541-1.772	0.336			
FIGO stage (I+II vs. III+IV)	1.609	1.192-2.376	**0.003**	1.891	1.233-2.835	**0.001**
Lymph node invasion (negative vs. positive)	1.557	1.112-2.197	**<0.001**	1.594	1.219-2.081	**<0.001**
Response to chemotherapy (sensitive vs. resistant)	0.197	0.002-0.392	**0.002**	0.563	0.142-1.428	**0.001**
